# Lessons learned from 100 failed mouse Langendorff experiments

**DOI:** 10.1016/j.xjon.2026.101582

**Published:** 2026-01-14

**Authors:** AlleaBelle Bradshaw, Caroline Tran, Jennifer S. Lawton

**Affiliations:** a Division of Cardiac Surgery, Department of Surgery, Johns Hopkins University, Baltimore, Md; b Johns Hopkins University, Baltimore, Md

**Figure:**
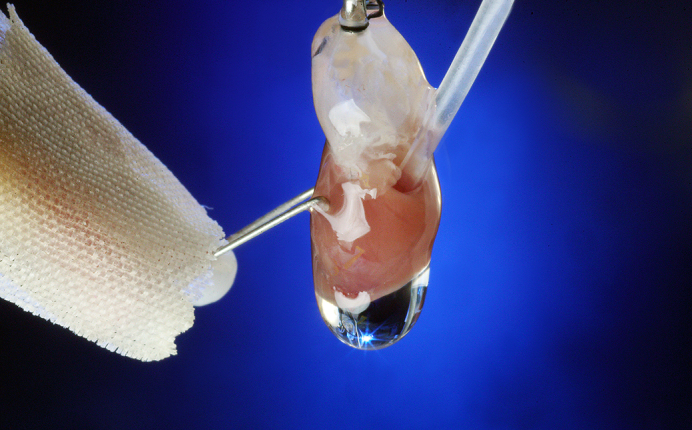
Mouse heart on a Langendorff column.

**Central Message**

Failures from mouse Langendorff experiments result in several transferrable skills, including technical proficiency, problem-solving, and knowledge of cardiac physiology.

**Perspective**

The Langendorff model has been important for advancing cardioprotection research, yet its technical demands often limit success. By outlining practical lessons from repeated failures, we provide guidance that may shorten the learning curve for others and improve experimental rigor in a model vital to cardiac surgery research.

The Langendorff model uses an isolated animal heart in an ex vivo perfusion system. With this model, one can study how the myocardium responds to a range of independent variables. The strength of the model is that all other variables that are not being studied can be removed or at least controlled, allowing a singular focus on the variable of interest. The uses of this model in cardiac surgery and cardiology are far-reaching, but include studying cardioplegia solutions, donor heart protection in transplant surgery, and recovery from myocardial infarction.^1^ Many animal models have been used with a Langendorff system, including mice, rats, rabbits, and pigs. Each animal model has pros and cons, including cost, size (and associated technical difficulty), and ease of care.

In our cardiac surgery laboratory, mouse hearts are used in a prolonged global ischemia (90 minutes) model of Langendorff perfusion (Figure 1).^2^ We have studied the cardioprotective efficacy of ATP-sensitive potassium (K_ATP_) channel openers including diazoxide.^3^, ^4^, ^5^, ^6^ Our work with this mouse model has led to successful large animal models and an early-phase human clinical trial (Safety and Feasibility of Hyperkalemic Cardioplegia With Diazoxide in Cardiac Surgery [CPG-DZX] Trial, clinicaltrials.gov NCT06308107).^2^^,^^7^, ^8^, ^9^, ^10^, ^11^ We continue to use the model to narrow experimental groups before use in large animal models with the ultimate goal of improving outcomes in humans.

**Figure 1:**
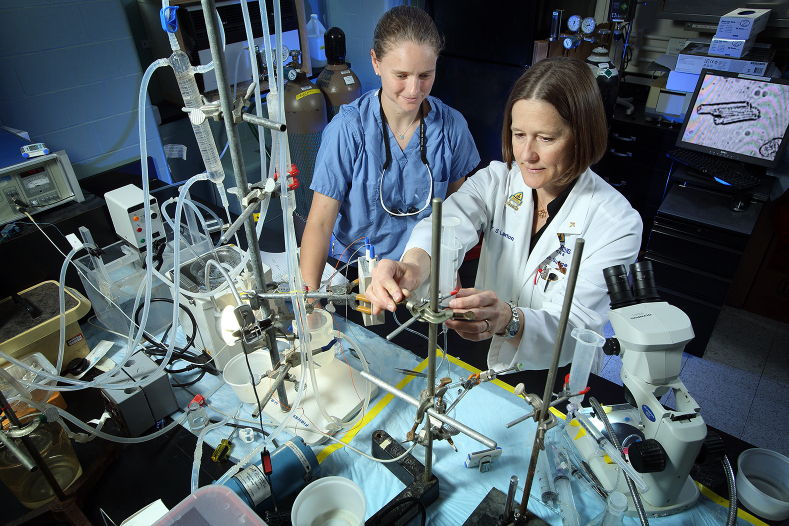
Langendorff perfusion in the Lawton laboratory. Drs Kathleen Clement (*left*) and Jennifer Lawton (*right*) preparing a Langendorff column for an experiment at Johns Hopkins Hospital. In this experiment, oxygenated perfusion solution is circulated by a centrifugal pump through bypass pump tubing before being delivered to the coronary arteries via an aortic cannula.

Our laboratory transitioned from the rabbit^12^, ^13^, ^14^, ^15^ to the mouse Langendorff model in an effort to investigate the individual K_ATP_ channel components involved in myocardial protection using genetic deletion.^7^ In addition, mice are relatively inexpensive and easy to care for, which make them suitable for the number of experiments needed to improve the power of the studies. Unfortunately, mouse hearts are small, making experiments extremely technically challenging. The mouse aorta is approximately 1.0 to 1.2 mm in diameter, and it is cannulated with a blunt-ended, 18-gauge needle. Performing this cannulation requires practice before consistent success. Success here often hinges on the operator's dexterity, patience, and, to be completely honest, willingness to emotionally detach from their failures.

The basic steps include rapid cardiectomy, cannulation of the aorta with the heart immersed in cold saline under a microscope, rapid transfer of the cannulated heart to the Langendorff column, and beginning perfusion with 37 °C modified Krebs-Henseleit Buffer (mKHB) solution (pH of 7.34-7.44). A water-filled silicone balloon is carefully inserted into the left ventricle (LV). This balloon is attached to a catheter and connected to a pressure transducer. Small epicardial pacing leads are attached to the heart and paced at 450 beats/min, only slightly faster than the operator's heart rate during canulation! The coronary flow is measured by an inline monitor in the Langendorff column to ensure consistent flow between experiments. After the heart is on the column with the balloon inserted and the pacing wires on, data collection begins. The heart is perfused for 30 minutes, and then volume is added to the balloon 5 times. This is done slowly, at 1-μL increments using a specialized syringe. The corresponding end-diastolic and developed pressures are recorded as a measure of contractility as the balloon volume increases. After baseline pressures are recorded, balloon volume is decreased back to baseline and the heart is arrested (perfusion and pacing stopped) for 90 minutes to exaggerate the global ischemic insult of cardiac surgery. Cardioplegia (depending on the treatment group and the specific experiment) is given just before the onset of ischemia. After the ischemic period, perfusion and pacing are resumed. After 30 minutes of reperfusion, a second pressure-volume curve is recorded. The difference between the baseline and reperfusion pressure is the indicator of the treatment's cardioprotective effectiveness.

Similar to the complex endeavor of cardiac surgery, many things in this process can go wrong. In fact, failure is so consistent that one might be tempted to consider it a design feature rather than a flaw. Even with all necessary supplies and equipment and a textbook-perfect Langendorff setup, success is far from guaranteed. There can be technical problems at cannulation or balloon insertion, equipment failures, or missed steps during the daily setup routine. Both identifying and rectifying the problems that lead to any given procedural failure can be difficult. Further, staying engaged with the project and the end goal can be challenging after encountering many failures.

For one author (A.B.), more than 86 “practice” experiments were performed before success became consistent enough to begin collecting meaningful data. Previous Langendorff researchers in our laboratory made the understandable choice not to diligently count their failures, so we cannot report others’ learning curves. The most common early failure point was aortic cannulation. It often exceeded 5 minutes, necessitating aborting the procedure at that point. Coronary flow issues were the next most frequent problem. Low flow was usually due to air embolism; conversely, unexpectedly high flow often signaled a loose ligature or a small tear in the aorta. Once these failures could be consistently avoided, new challenges emerged in maintaining myocardial viability and obtaining accurate physiologic measurements. These experiences informed the following lessons learned after approximately 100 failed Langendorff experiments (Figure 2).

**Figure 2:**
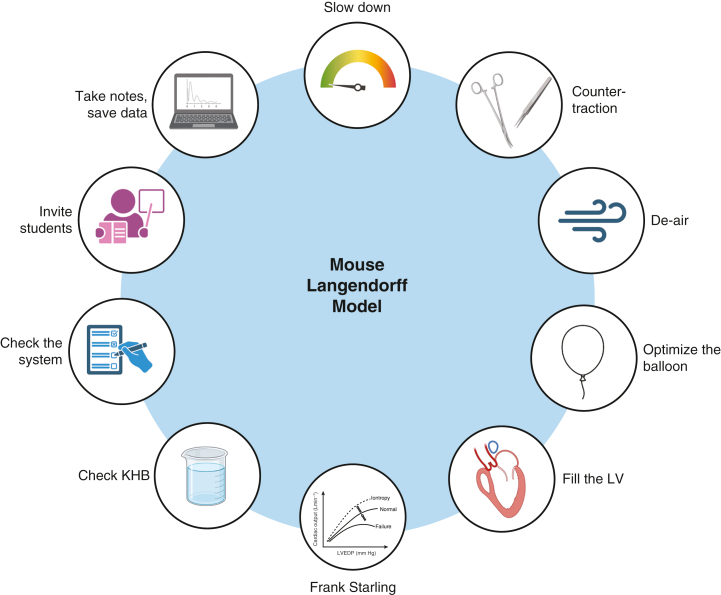
Lessons and reminders for success in a mouse Langendorff model. Using lessons learned from many failures with the technically challenging mouse Langendorff model, we summarize ways to improve the chances of success. Figure created using Biorender.com. *KHB,* Krebs-Henseleit Buffer; *LV,* left ventricle.

### 1. Slow is Smooth, Smooth is Fast

Although this is a well-known mantra from surgical training, it has never been truer than when trying to cannulate the mouse aorta or insert the balloon. When cannulating, time is of the essence because prolonged time to cannulate increases myocardial injury and could be considered preconditioning that will influence the study results. This concept mirrors clinical cardiac surgery, such as when the surgeon performs a coronary anastomosis as quickly as possible to minimize crossclamp time. The goals should be the same when performing the mouse aorta cannulation as with an anastomosis: efficiency and precision. Ironically, functioning under the assumption that hurried movements leads to better efficiency is a mistake. Hurried movements are imprecise, resulting in mistakes that require time-consuming corrections. Focus on smooth, deliberate, methodical movements; although these movements may feel slower, they result in a faster cannulation. In other words, instead of seeking to be fast, seek to be steady and find a rhythm.

### 2. Counter-traction: Using Instruments Effectively

The most intimidating step of the Langendorff experiment is aortic cannulation. This is done while the heart and cannula are submersed in cold mKHB solution. After placing the aorta on the cannula, a 6-0 silk suture is tightened around the aorta. The suture is already there with a pre-tied first loop, it just needs to be tightened, and then additional loops must be tied and tightened. Tightening the first loop is difficult because it is often necessary to use one hand to constantly hold the aorta on the cannula (depending on the length of the aorta). How does one tie down a suture with only one remaining one hand? Using a soft bulldog to hold tension with the suture on one side of the cannulation works well to solve this problem. Thus, you can pull on one side of the suture with the one hand and grasp the aorta with the other hand. This reinforces another surgical principle, efficiency of motion (being smooth and steady) is necessary but not sufficient. One also must use the right instruments.

### 3. Air is the Enemy, but Only if it Moves

If any air gets into the aortic cannula or the heart, air can enter the coronary arteries, resulting in insufficient coronary flow. In such cases, the experiment typically must be aborted because there is no reliable way to de-air the system. Using a large syringe to de-air the Langendorff column retrograde with warm mKHB just before starting the experiment can prevent air from collecting near the bottom of the column, where it can enter the heart. If the air pocket is “locked” up in the middle of the column (often at the level of the filter), it is harmless. In this model, air bubbles are like bad ideas: harmless if stationary, catastrophic when allowed to circulate.

### 4. Optimize the Balloon Before Starting

Our protocol requires the creation and cannulation of a silicone balloon.^16^ After securing it to a short piece of tubing, the balloon is filled with water. Proper setup here is essential for obtaining accurate LV pressures, which are the primary outcomes. One potential problem is a loose tie at the balloon neck that allows water to leak around the balloon. (A familiar surgical truth: Air knots are never good.) Two tightly placed 6-0 silk ties have proven the most reliable solution. A second problem is air entrainment in the balloon tubing, which can be impossible to get out. A trace amount can be inconsequential, but a large volume or several small bubbles can dampen the pressure signals, resulting in inaccurate pressures. Air is most commonly introduced during routine zeroing, when the system is briefly opened, so this step warrants particular caution.

### 5. The Balloon Must Fill the LV

After becoming reasonably consistent at cannulation and balloon insertion, the challenges of data collection become apparent. For the LV balloon to transduce pressures accurately, the balloon must fill the ventricle. This is different from an in vivo heart that is full of blood. A catheter sitting in the LV of a heart that is already full can transduce pressures. However, the ex vivo LV (assuming one is not using a working-mode Langendorff) is full of air, so the balloon must be full to transduce accurate pressures. If the balloon must be deflated so that it can fit through the tiny hole in the left atrium and traverse the mitral valve into the LV, then immediately add enough air to allow it to fill the ventricle before interpreting the pressures.

Once the balloon is full, it also must be positioned well. If the heart is otherwise functioning appropriately (pacing well, coronary flow within range, good pressure waveform) and the balloon is full, but the pressures still seem off, consider making minor adjustments to the balloon position. Such adjustments can improve the position of the balloon in the LV, so that it transduces accurate pressures.

### 6. Remember the Frank Starling Law

Although it is important to make sure the balloon fills the LV, overfilling it creates another problem. The Frank Starling Law states that within a physiologic range, stretching the cardiac muscle will result in greater ventricular stroke volume or cardiac output. However, the Frank Starling law also states that at a certain point, more stretching no longer results in more output; there is a plateau. Thus, in the mouse Langendorff experiments, the balloon needs to be filled to approximate the physiologic volume of the mouse LV. If the balloon is overfilled, then increasing the balloon volume does not result in the expected pressure-volume loop.

### 7. Check the Perfusion Solution

If the balloon is inserted and filled optimally, initial pressures should fall within reasonable ranges, ensuring good perfusion. We expect an initial LV end-diastolic pressure of approximately 2.5 mm Hg and a developed pressure of approximately 30 mm Hg. If the pressures are acceptable initially but then heart failure develops over the first 5 to 10 minutes of perfusion, the problem is likely with the perfusion solution. The mKHB solution is supposed to be approximately a pH of 7.4 after bubbling with 95% O_2_ and 5% CO_2_. It is also supposed to have a calcium concentration of approximately 1.4 mmol/L. However, these numbers are not guaranteed just because the recipe is followed. Several factors can affect the pH of the solution, so always check it before starting. If the pH is off, correct it with HCl or NaOH, or just make new solution. Additionally, confirm the purity of the reagents being used. Calcium chloride dihydrate reagent can absorb water over time, resulting in low calcium concentrations. Hypocalcemia causes inadequate contractility and subsequent heart failure. Checking the pH and concentrations of solutes like calcium can be the difference between success and continued frustration.

### 8. Respect the System's Complexity

In the Langendorff model, success relies not only on surgical skill but also on relentless attention to particulars. Attention to every clamp, warmer, hardware element, stopcock, and tubing connection requires disciplined focus. Use a checklist to create reliable system to ensure system functionality. This checklist can transform the procedure from unpredictable and chaotic to methodical and purposeful, improving success rates. Notably, the system has many parallels to the cardiopulmonary bypass circuit in the cardiac surgery operating room, which the surgeon must be intimately familiar with.

Failures with the system have led to hard-earned lessons about different components. Forgetting to unclamp heater coil tubing after cleaning and reassembling the system can lead to multiple hearts failing before realizing the perfusion solution is running in cold. As it turns out, clean, well-maintained equipment is only useful if it is also operational. There are several other things to check on the system before starting or early during perfusion, each of which has caused failures: aortic cannula seated well (it can fall off), pressure transducer zeroed and calibrated, water bath temperature accuracy (use a thermometer to confirm), adequate O_2_/CO_2_ in tank to complete experiment, and balloon integrity. These small oversights turn into big problems.

### 9. Invite Students

When medical or college students visit the laboratory to observe or assist, they bring a fresh excitement. Their enthusiasm, which has not been tempered by countless prior failures, is motivating. Even when an experiment fails, they often remain optimistic. This enthusiasm leads to them reframing failures as progress, resulting in innovation and problem-solving. Participation in experiments is also highly valuable for students, who gain exposure to technical challenges, practice real-time problem-solving, and deepen their understanding of cardiac physiology. Recently, an undergraduate student (C.T.) applied for her own independent funding to support a new arm of experiments with the mouse Langendorff model. She also contributed to this article.

### 10. Take Careful Notes and Save Data

The value of an extremely detailed laboratory notebook cannot be overstated. First, the daily process of writing out the steps (and missteps) after each experiment requires thoughtful reflection, which often leads to new realizations when first learning the model. It is a form of “debriefing.” Second, detailed notes of both successes and failures make it possible to remember what happened days or weeks ago, preventing repetition of the same mistakes. It is also helpful to look back at notes from prior researchers. You may not realize you have a problem with your calcium concentration without reviewing a laboratory notebook from years prior, in which the researcher had recorded the pH and electrolyte concentrations of each mKHB solution. By contrasting the properties of the solutions, it becomes obvious that the calcium levels are different despite using the same recipe.

For hemodynamic data that are recorded digitally, save files in a secure network, not just at the local desktop. This includes successful and failed experiments. Even a failed experiment can be helpful to review later. No data are ever wasted if someone can learn from it, but data that are not backed up are data already consigned to oblivion.

## Conclusions

The Langendorff model has been used for more than 130 years, since 1895.^1^ Despite this long history, the technical challenges of the model, especially for small animals, remain. Failed experiments should not be viewed as wasted efforts but as opportunities to gain insight into subtle aspects of perfusion, cannulation, and physiology that often go unnoticed in successful trials.

The discipline required mirrors the elements of cardiac surgery itself: careful preparation, respect for physiology, and the ability to troubleshoot under pressure. Technical habits developed at the Langendorff bench translate naturally to clinical practice, from managing perfusion circuits to interpreting real-time hemodynamic signals. Although the model may appear esoteric at first glance, its lessons are broadly applicable and deeply valuable. Embracing the inevitable failures makes the system more intuitive, strengthens experimental rigor, and ultimately improves the quality of both the science and the surgeon.

## Conflict of Interest Statement

The authors reported no conflicts of interest.

The *Journal* policy requires editors and reviewers to disclose conflicts of interest and to decline handling or reviewing manuscripts for which they may have a conflict of interest. The editors and reviewers of this article have no conflicts of interest.
